# Use of bacterial whole-genome sequencing to investigate local persistence and spread in bovine tuberculosis

**DOI:** 10.1016/j.epidem.2015.08.003

**Published:** 2016-03

**Authors:** Hannah Trewby, David Wright, Eleanor L. Breadon, Samantha J. Lycett, Tom R. Mallon, Carl McCormick, Paul Johnson, Richard J. Orton, Adrian R. Allen, Julie Galbraith, Pawel Herzyk, Robin A. Skuce, Roman Biek, Rowland R. Kao

**Affiliations:** a Boyd Orr Centre for Population and Ecosystem Health, Institute for Biodiversity Animal Health and Comparative Medicine, University of Glasgow, Glasgow, UK; b School of Biological Sciences, Queen's University Belfast, Belfast, Northern Ireland, UK; c Veterinary Sciences Division, Agri-Food and Biosciences Institute, Stormont, Belfast, Northern Ireland, UK; d The Roslin Institute, University of Edinburgh, Edinburgh, UK; e Glasgow Polyomics, College of Medical Veterinary and Life Sciences, University of Glasgow, Glasgow, UK

**Keywords:** Bacterial evolution, Livestock disease, Molecular epidemiology, *Mycobacterium bovis*, Phylogeography

## Abstract

*Mycobacterium bovis* is the causal agent of bovine tuberculosis, one of the most important diseases currently facing the UK cattle industry. Here, we use high-density whole genome sequencing (WGS) in a defined sub-population of *M. bovis* in 145 cattle across 66 herd breakdowns to gain insights into local spread and persistence. We show that despite low divergence among isolates, WGS can in principle expose contributions of under-sampled host populations to *M. bovis* transmission. However, we demonstrate that in our data such a signal is due to molecular type switching, which had been previously undocumented for *M. bovis*. Isolates from farms with a known history of direct cattle movement between them did not show a statistical signal of higher genetic similarity. Despite an overall signal of genetic isolation by distance, genetic distances also showed no apparent relationship with spatial distance among affected farms over distances <5 km. Using simulations, we find that even over the brief evolutionary timescale covered by our data, Bayesian phylogeographic approaches are feasible. Applying such approaches showed that *M. bovis* dispersal in this system is heterogeneous but slow overall, averaging 2 km/year. These results confirm that widespread application of WGS to *M. bovis* will bring novel and important insights into the dynamics of *M. bovis* spread and persistence, but that the current questions most pertinent to control will be best addressed using approaches that more directly integrate WGS with additional epidemiological data.

## Highlights

• We performed whole genome sequencing (WGS) of Mycobacterium bovis for a single molecular (VNTR) type.
• Under-sampling of one lineage was caused by switching between VNTR-types.
• Pairwise SNP distances showed a weak genetic isolation by distance pattern.
• Bayesian phylogeographic inference was feasible despite a low substitution rate.
• WGS studies of M. bovis need to account for slow evolution and molecular type switching.

## 1 Introduction

The increasing availability of bacterial whole-genome sequence (WGS) data now makes it possible to generate sequence datasets for whole bacterial pathogen populations at high sampling densities. Such comprehensive sequencing has yielded impressive advances in outbreak investigation (Eyre et al., 2013, Harris et al., 2010, Walker et al., 2012), and provided new insights into both spatial dissemination (Gray et al., 2011, Holden et al., 2013) and the complexities of multi-host pathogen systems (Mather et al., 2013, Viana et al., 2014). However, even at the genomic scale the rates of evolutionary change estimated for bacteria can be substantially lower than those commonly seen in rapidly evolving pathogens such as RNA viruses (Biek et al., 2015, Bryant et al., 2013b). The extent to which slow evolution constrains the type and scale of epidemiological processes that can be resolved for bacterial pathogens, and which analytical approaches are most appropriate to deal with this, remains unclear for many systems.

*Mycobacterium bovis* is one of a group of closely related bacteria which includes the primary cause of human tuberculosis, *M. tuberculosis*, a pathogen estimated to evolve at a rate of around 0.3–0.5 mutations per genome per year over epidemiological timescales (Bryant et al., 2013b, Walker et al., 2012). *M. bovis* is the causative agent of bovine tuberculosis (bTB), an important disease of cattle and other mammals including man. Herd-to-herd movements of infected cows among farms (Gilbert et al., 2005, Green et al., 2008) and infection in Eurasian badger (*Meles meles*) populations (Delahay et al., 2001, Gallagher and Clifton-Hadley, 2000) have both been implicated in the spread of bTB in Britain and Ireland. While much attention has focused on the relative roles of badgers and cattle in the maintenance of bTB, recent studies emphasising the importance of cattle have highlighted the continuing need for a deeper understanding of the role of cattle-based transmission (Brooks-Pollock et al., 2014, Donnelly and Nouvellet, 2013).

Molecular typing of *M. bovis* isolates based on repeated genetic elements has been advocated for some time to aid in the epidemiology and control of bTB (Cousins et al., 1998, Skuce and Neill, 2001), and in Britain and Ireland these typing methods have shown that *M. bovis* molecular types are maintained within well-defined geographic clusters (Skuce et al., 2010, Smith et al., 2006). While such molecular typing has proved useful for identifying local clustering on larger scales, their power to discriminate within-cluster events involved in fine-scale persistence and spread of bTB is limited.

In a previous study, Biek et al. (2012) established the potential of bacterial WGS in investigating the epidemiology of bTB at a local (i.e., farm-to-farm) scale. By sequencing 30 bacterial isolates from a spatially dense cluster of bTB cases within one recently emerged *M. bovis* molecular type (VNTR-10) in Northern Ireland (NI), the study demonstrated (i) close relatedness of bacteria isolated from cattle and badgers, (ii) persistence of bacterial lineages on the same farm, and (iii) that genetic similarity between isolates correlated with geographic distance between sampling locations. The study also showed that, due to slow evolution, even WGS is unlikely to provide sufficient resolution to resolve transmission at the animal-to-animal scale for *M. bovis*, similar to findings in human tuberculosis (Bryant et al., 2013b, Roetzer et al., 2013, Walker et al., 2012), and is more suited to do so at the between-farm scale.

While providing a proof of concept, this previous study was targeted towards a subsample of VNTR-10 infected cattle within a small (approx. 5 km) spatial radius. This spatially restricted sampling precluded a more systematic investigation of processes occurring on a wider, population level scale within the bacterial strain, including the potential identification of under-sampled reservoirs, the rate and mode of spatial spread, and transmission links between bTB breakdowns. A herd breakdown is defined as the period during which movements of cattle out of a herd are restricted due to the detection of bTB in the herd, starting at the detection of one or more infected animals (either through the tuberculin skin test or through abattoir surveillance for bTB lesions), and ending when the herd has undergone two consecutive negative whole-herd tests at least 60 days apart, or a single negative test where the breakdown was not laboratory confirmed.

Here, we extend the analysis of the Biek et al. (2012) study by examining WGS data from all 145 available VNTR-10 isolates in NI since 2003. In NI all cattle herds are tested for bTB on an annual basis, and for over a decade *M. bovis* isolates cultured from test-positive cattle have been extensively typed and stored. These archived samples therefore gave us the opportunity to target a genetically defined sub-population of *M. bovis* (VNTR-10) for high-density sampling with respect to cattle infections, although VNTR-10 infections in any other population would not be accessible through this sampling strategy. To gain insights into the mode and rates of transmission, we used intensive sampling and WGS of *M. bovis* isolates from cattle to address the following questions:

(1) Does WGS of VNTR-10 isolates from cattle indicate contributions from another host population which is under-sampled under the above sequencing strategy?
(2) Does the genetic relatedness between sequenced isolates correlate with recorded movements and/or with spatial distance between premises?
(3) What are the rate and mode of *M. bovis* dispersal across the landscape at the between-breakdown scale as determined by WGS?

## 2 Materials and methods

### 2.1 Molecular-typing of *M. bovis* in NI

In NI, *M. bovis* isolates have been stored and typed since the early 2000s using spoligotyping, more recently combined with Variable Number Tandem Repeat (VNTR) typing, to differentiate molecular types (Skuce et al., 2005). Spoligotyping gives a relatively coarse-grained discrimination of the *M. bovis* population, and is based on the presence or absence of multiple spacer oligonucleotides within the direct repeat region of the genome (Kamerbeek et al., 1997). VNTR-typing indexes the number of short nucleotide repeats present at several VNTR loci identified within the mycobacterial genome (Mazars et al., 2001), and provides greater discriminatory power than spoligotyping alone, although with a relatively higher chance of homoplasy (i.e., separate lineages converging on the same molecular type). In NI between 2003 and 2008 one *M. bovis* isolate was VNTR-typed and stored from each herd breakdown for which *M. bovis* was isolated using a panel of 7 VNTR loci optimised for this population of *M. bovis* (Skuce et al., 2010), while from 2009 onwards bacteria were VNTR-typed and stored from all culturable cattle cases, therefore resulting in more intensive sampling in recent years. In addition to these cattle isolates, *M. bovis* has also been typed by spoligotyping and VNTR-typing and archived when isolated from a survey of badgers killed on the roads in NI (Abernethy et al., 2011).

### 2.2 Bacterial samples and sequencing

Cultures of *M. bovis* were isolated from bovine granulomatous tissue using conventional methods (Skuce et al., 2010). Confirmed isolates were grown on LJ slopes to single colonies, following which single colonies were grown up and DNA was extracted using standard CTAB and solvent extraction protocols (Van Soolingen et al., 2001). A total of 144 VNTR-10* M. bovis* isolates were included in this study, from 66 herd breakdowns (see earlier definition) occurring in 51 herds between 1996 and 2011. The WGS dataset consisted of the raw reads from 31 VNTR-10 samples originally sequenced in the preceding study (26 cattle and 5 badger isolates; Biek et al., 2012), in addition to 114 VNTR-10 samples (113 cattle and 1 badger isolate) sequenced for the first time in this study. VNTR-10 is located predominantly within the Newtownards area of NI (178/195 VNTR-10 infections recorded between 1996 and 2011 were from the Newtownards district veterinary office), and statistical comparison to other VNTR types confirms that VNTR-10 is generally representative of strains circulating in this area (see supplemental information). Details of the accession numbers for the raw sequencing reads are given in Table S1.

**Fig. 1:**
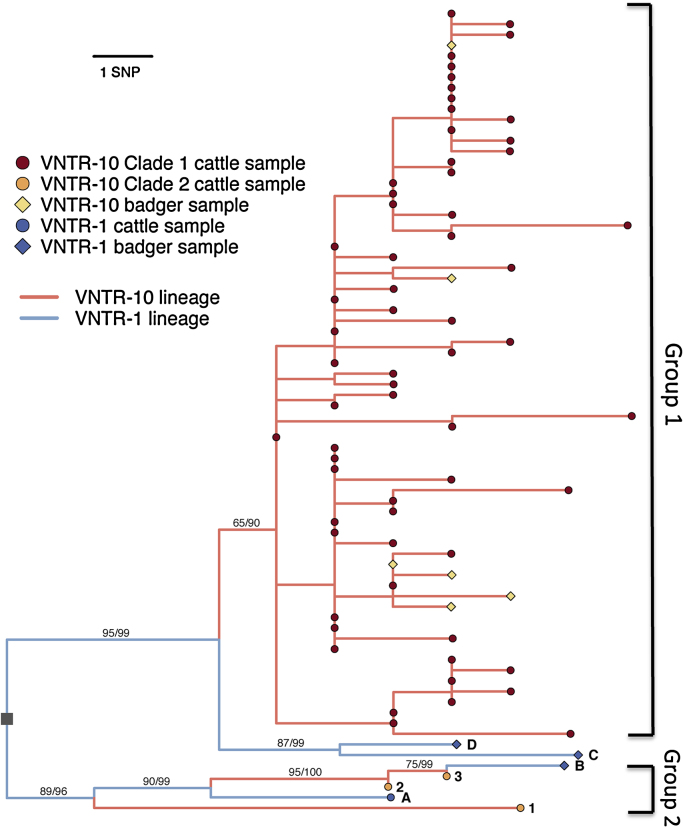
Maximum Likelihood phylogeny of VNTR-1 and -10 isolates subsampled to one sequence per outbreak and rooted on the VNTR-4 isolate and *M*. *bovis* reference sequence (Garnier et al., 2003) (not shown; the node used to root the phylogeny is indicated by a grey square). Tip colours give details of the samples: red circles are Group 1 VNTR-10 cattle isolates, orange circles (numbers 1–3) are Group 2 VNTR-10 cattle samples; yellow diamonds are VNTR-10 badger isolates; blue circle (A) is the VNTR-1 cattle isolate, blue diamonds (B–D are VNTR-1 badger isolates. Branch colours give the likely VNTR-type of each branch, assuming the most recent common ancestor of the group was VNTR-1. Branch labels show the statistical support for selected nodes: the left-hand value indicates percentage bootstrap support from a maximum likelihood phylogeny generated for these isolates, and the right-hand value shows posterior probability of the node in the Bayesian phylogeny generated for these isolates. (For interpretation of the references to colour in this figure legend, the reader is referred to the web version of this article).

**Fig. 2:**
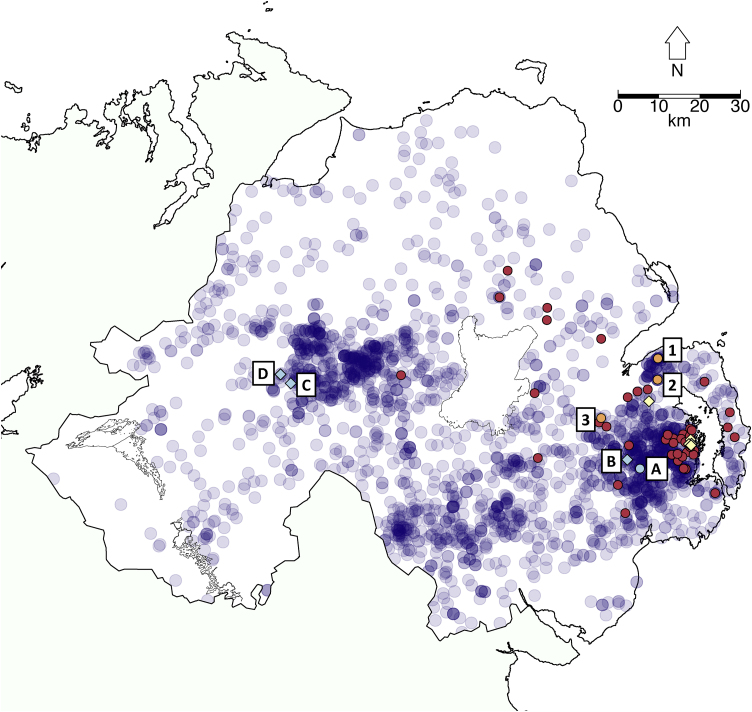
Map of Northern Ireland showing origins of sequenced samples. Red circles are Group 1 VNTR-10 cattle isolates; orange circles (samples 1-3) are Group 2 VNTR-10 cattle samples, yellow diamonds are VNTR-10 badger isolates, light blue diamonds (badger) and circle (cattle) (samples A–D) are VNTR-1 isolates, and dark blue transparent circles show locations of all other VNTR-1 isolates. (For interpretation of the references to colour in this figure legend, the reader is referred to the web version of this article).

To provide broader evolutionary context we additionally sequenced five samples from VNTR-types thought to be ancestral to VNTR-10, namely four isolates of VNTR-1 (1 cattle isolate (sample A), and 3 badger isolates (samples B–D, Fig. 1, Fig. 2)), and one cattle isolate of VNTR-4. VNTR-1 is thought to be the direct ancestor of VNTR-10 based on the following observations: VNTR-1 and VNTR-10 are separated by a single tandem repeat difference; VNTR-1 has been recorded at a high and approximately stable prevalence in NI since routine VNTR-typing commenced (Skuce et al., 2010, Skuce et al., 2005), whereas VNTR-10 has been found in low but increasing numbers suggestive of a newly emerged strain; VNTR-1 is found across a wider spatial range than VNTR-10 (Fig. 2); and a minimum spanning tree of all NI VNTR-types within spoligotype SB0140 shows that VNTR-1 is basal compared to VNTR-10 (Fig. S1). The VNTR-4 type differs from VNTR-1 by two tandem repeats (Table S2), however it is not possible to determine which of them is ancestral. Sequencing was performed at Glasgow Polyomics at the University of Glasgow using the Illumina IIx platform, with the exception of VNTR-1 samples B-D, sequenced using an Illumina MiSeq.

### 2.3 Sequence analysis

Full details of the bioinformatics workflow are provided in the Supplementary Information. Briefly, reads were trimmed and mapped to the *M. bovis* reference genome (GenBank accession number BX248333; Garnier et al., 2003) using BWA (Li and Durbin, 2009). Variants were identified using SAMtools (Li et al., 2009) and filtered on base quality, mapping quality, heterozygosity, proportion of samples with high quality calls at each site, clustering of variant loci, and location relative to repeat regions of the genome. The resulting variant sites were concatenated for each isolate, giving the genetic sequences used for downstream analyses.

A maximum likelihood phylogeny was generated in PhyML v3.0 (Guindon and Gascuel, 2003) under the Jukes Cantor model of nucleotide substitution, including the *M. bovis* reference sequence as outgroup, and evaluating statistical support for individual nodes based on 1000 non-parametric bootstraps. A Bayesian phylogeny was generated in MrBayes (Ronquist and Huelsenbeck, 2003) under the Jukes Cantor model, also including the *M. bovis* reference sequence, and was run for 10^6^ MCMC iterations at which point the standard deviation of split frequencies was below 0.01. Raw pairwise single nucleotide polymorphism (SNP) differences between sequenced samples were calculated in MEGA 5 (Tamura et al., 2011), using pairwise deletions in the event of missing data. Due to the increased level of sampling from 2009 onwards mentioned above and the low levels of within-breakdown diversity (see Results), further analyses were restricted to one representative sample per herd breakdown.

### 2.4 Comparing genetic and epidemiological relationships between breakdowns

In NI, detailed information on the cattle population and movements between herds and bTB test results is recorded by the Department of Agriculture and Rural Development (Houston, 2001). All direct cattle movements between herds with VNTR-10 samples represented in our dataset (66 sequenced breakdowns and 12793 individual cattle movements) were made available, and were combined with the location and date of each sequenced sample using SQL (MySQL v5.5.29; www.mysql.com). All further analyses were conducted in R v3.0.1 (R Core Team and R Development Core Team, 2014) unless otherwise stated.

A Mantel test was conducted in the R package ecodist (Goslee and Urban, 2007, Lichstein, 2007) to assess correlation between spatial and genetic distance between Group 1 breakdowns, using 10000 permutations to assess significance (see results and Fig. 1 for definition of Group 1). To further confirm this correlation, a multiple regression on distance matrices was also carried out using the R package ecodist (Lichstein, 2007), assessing the correlation between the matrix of pairwise SNP differences and the matrix of geographic distances between Group 1 breakdowns, and using 10000 permutations to assess significance.

For each sequenced Group 1 breakdown, we recorded whether it was linked to one or more other sequenced breakdowns by potentially infectious cattle moving directly into the original breakdown herd (“movement links”). We considered movements occurring within 2, 5, and 10 years prior to the official start of each breakdown. Few direct movement links were identified within the 2- and 5-year windows (15 and 53 links, compared to 102 for the 10-year window). We therefore included all movements identified within the most conservative window of 10 years in our analysis. For each sequenced breakdown, we identified any movement links to that breakdown from any other sequenced breakdown. Where links were present, we recorded the minimum pairwise SNP difference between the linked premises, as the minimum SNP difference is most likely to represent a direct transmission event if one exists between breakdowns.

To assess the effect of short-distance transmission mechanisms in a more targeted manner, we identified pairs of sequenced herds located within 2 km and 5 km of another sequenced herd. This choice was motivated by the observation that the vast majority of badger movements fall within 5 km, although larger distances are occasionally recorded (Byrne et al., 2014, Pope et al., 2006). We note however that transmission over short distances may be driven by various mechanisms and is not necessarily restricted to badgers. For each sequenced breakdown, we identified the presence of other sequenced breakdowns linked through spatial proximity at these distances, and again recorded the minimum number of SNPs separating it from other spatially-linked breakdowns.

A higher number of epidemiological links between breakdowns is likely to result in a lower minimum SNP differences between breakdowns due to the increased number of comparisons, and therefore the distributions of minimum SNP differences are not directly comparable between different types of links. To statistically assess the significance of the association between epidemiological links and minimum SNP differences, the distribution of the expected number of SNP differences was simulated for movement and proximity links under a null hypothesis of no association between the presence of a link and genetic relatedness between breakdowns. The number of links to each sequenced breakdown, identified above, was kept but these links were effectively “rewired” by permuting the matrix of linked outbreaks and thus randomising the breakdowns that each sequenced breakdown was linked to, and the minimum SNP difference was again calculated for each breakdown. This was repeated 10,000 times for each type of link (direct recorded movements within 10 years of breakdowns and spatial proximities of 2 km and 5 km) to generate null distributions for comparison to the observed distributions.

To formally compare the null simulations with the observed distribution of minimum SNP differences between linked outbreaks, a goodness of fit test was carried out. We generated 10,000 realisations of a multinomial distribution, with sample size equal to the number of linked breakdowns, and the probability of each category proportional to the expected value of each category (taken as the mean of the values from the null simulations). A Chi-squared test statistic was then calculated for each of these multinomial distributions. This gave a distribution of simulated test statistics to which the Chi-squared statistic calculated from the observed data was compared. A *p*-value was estimated as the proportion of the null Chi-squared statistics that were greater than the observed Chi-squared value.

### 2.5 Phylogeographic inference

To quantify the spread of *M. bovis* across the landscape, continuous phylogeographic models were applied using the Bayesian phylogenetic program BEAST v1.7.4 (Drummond et al., 2012, Lemey et al., 2010). This analysis was restricted to the VNTR-10 clade containing the majority of isolates (Group 1, see Results and Fig. 1), using one representative sample per breakdown. A strict Brownian model of spatial diffusion was compared to a relaxed model allowing diffusion rates to vary among branches, with rates drawn from a Cauchy distribution (see Supplemental Information). A relaxed model with branch rates drawn from a gamma distribution was also tested but failed to converge. Models were run for 5 × 10^8^ iterations, assessed for convergence in Tracer, and model fit evaluated based on log Marginal Likelihood Estimates (MLE) generated by using path-sampling and stepping-stone sampling in BEAST (Baele et al., 2012). Posterior trees for the best fitting model were combined to find and annotate the Maximum Clade Credibility (MCC) tree. Node locations, branch lengths, and branch-specific rates of geographic dispersal were extracted and evaluated for the MCC tree.

Given that the molecular clock rate of *M. bovis* and other closely related mycobacteria has been shown to be slow and variable (Biek et al., 2012, Bryant et al., 2013b), it was uncertain whether these data would contain enough genetic signal to accommodate phylogeographic analyses. To test this, we simulated a homogeneous spatial diffusion process along the MCC phylogeny generated above, guided by empirical rates, generating a set of spatial coordinates for sampled sequences under a set rate of spatial diffusion along the existing phylogeny. We then evaluated whether phylogeographic analysis in BEAST, using the settings described above, using the simulated coordinates and observed sequences and sampling dates as input, could recover the originally specified diffusion rate for each of 100 simulations.

## 3 Results

### 3.1 VNTR-10 isolates

Among the intensively sampled VNTR-10 isolates, genetic divergence was low; averaging 6.4 SNPs (range 0–19) over the whole group of 145 sequenced VNTR-10 isolates. Despite limited divergence, the group contained 39 shared polymorphisms resulting in a non-star-like phylogenetic structure (Fig. 1, showing one sample per herd breakdown).

Regressing genetic distance from the root of the phylogeny against sampling date revealed a moderate positive correlation (*R*^2^ = 0.32), indicative of a molecular clock signal within these data (Firth et al., 2010).

Average diversity for sequenced isolates from within the same herd breakdowns (including two breakdowns which were polyphyletic) was low, with mean 0.69 SNPs and range 0–4 SNPs. This was considerably lower than the average minimum SNP differences between different VNTR-10 breakdowns (mean 4.73 SNPs, range 0–17 SNPs). Multiple samples per breakdown were only available for 19 of the 66 VNTR-10 breakdowns. Given the low level of within-breakdown divergence and the fact that multiple samples per breakdown were only available after 2008 (see Materials and methods), we chose to use one representative sequence per herd breakdown for further analysis in order to focus on the dynamics of *M. bovis* spread at the between-breakdown scale. The full phylogeny, including all samples described here, is shown in Fig. S2.

Both Bayesian and Maximum Likelihood phylogenies indicated that the majority of VNTR-10 sequences fall into a single main group, hereafter referred to as Group 1, including 126 samples from 63 breakdowns, and including the six badger samples (Fig. 1). Nineteen other VNTR-10 samples, belonging to three individual breakdowns (represented by samples 1, 2, and 3, Fig. 1) were not positioned within Group 1 and instead clustered with two of the VNTR-1 samples (samples A and B, Fig. 1, Group 2). Both Group 1 and Group 2 isolates were defined by multiple unique SNPs, however statistical support for the nodes defining these two lineages was not consistently high (bootstraps of 65 and 89 respectively, and posterior probabilities of 90 and 96, Fig. 1).

Despite the much larger number of Group 1 samples, these two sets of VNTR-10 samples were similar in terms of maximum genetic divergence among isolates (Group 1 samples: 15 SNPs; Group 2 samples: 13 SNPs). Within Group 1, the average pairwise distance was low (mean: 4.4 SNPs), many isolates were genetically indistinguishable, and the majority of mutational steps were represented by one or more sequenced isolates. These observations are indicative of a population that has been comprehensively sampled, consistent with our expectations based on sampling all VNTR-10 outbreaks detected over the study period. In contrast, sequences from the Group 2 VNTR-10 breakdowns were non-identical, more divergent from each other (mean: 8.5 SNPs) and many of the intermediate mutational steps were not represented in the sequenced samples. This suggests that comprehensive sampling of the lineage represented by these isolates had not been achieved.

To evaluate whether these differences might simply be due to sampling, 1000 random subsamples of three sequences (matching the number of Group 2 VNTR-10 breakdowns) were generated from Group 1 isolates. The observed pairwise differences between Group 2 VNTR-10 isolates (1, 12, and 13 SNP differences) were at the upper end of the simulated distribution of pairwise distances among subsamples (mean 4.16 SNPs, range 0–12 SNPs; Fig. S3). This suggests that Group 2 isolates were sampled from a bacterial population that is at least as large as, or even larger than Group 1 (assuming no significant differences in evolutionary rates between the two lineages), but that a lower proportion of this population has been sampled and sequenced compared to Group 1, despite the consistently high sampling effort for VNTR-10 outbreaks in cattle.

Given that the Group 2 isolates appeared to come from a population with different characteristics compared to Group 1, and given that these comprised only three sampled VNTR-10 breakdowns, we restricted all further analyses to Group 1 samples unless otherwise indicated.

### 3.2 VNTR-types 4 and 1

As anticipated, the single VNTR-4 isolate was genetically highly distinct from the VNTR-10 group, differing by an average of 89 SNPs (Fig. S2). In contrast, VNTR-1 isolates failed to form a separate clade, as would have been expected for a separate VNTR-type, and instead were found to be nested within the VNTR-10 group. The two VNTR-1 isolates originating in the same geographical area as the majority of the VNTR-10 samples (samples A and B, Fig. 2) were found to cluster with VNTR-10 Group 2 samples (Fig. 1), while those originating from outside the VNTR-10 range formed a sister group to VNTR-10 Group 1 (Fig. 1, samples C and D, see also ‘Badger isolates’ below).

### 3.3 Badger isolates

As discussed by Biek et al. (2012) using a subset of the data described here, VNTR-10 *M. bovis* isolates from badgers and cattle were highly similar genetically, with a minimum distance of 0–3 SNPs to the most closely related cattle isolate (Fig. 1), suggestive of recent transmission links between badger and cattle. While the current study included only one additional VNTR-10 badger isolate, our high density sampling of VNTR-10 cattle infections also showed that the cattle isolates most closely related to those from badgers were all found within very close spatial proximity (<1.5 km) to the locations of these badger isolates.

Of the three newly sequenced VNTR-1 badger isolates, two samples (C and D) originated from an area outside the distributional range of VNTR-10, and approximately 100 km from the area where the majority of VNTR-10 isolates were located (Fig. 2). These two isolates were closest to VNTR-10 Group 1 sequences, separated by a minimum genetic distance of 5 SNPs (Fig. 1). As described above, the other VNTR-1 badger isolate (sample B) originated from the same area as the majority of the VNTR-10 samples (Fig. 2), and clustered with Group 2 VNTR-10 isolates (Fig. 1)

### 3.4 Molecular clock rate

Bayesian evolutionary analysis revealed an evolutionary rate of 0.2 substitutions per genome per year (95% HPD 0.1–0.3), estimating the time of the most recent common ancestor of the VNTR-10 group as a whole as 1974 (1954–1989), and the time of the most recent common ancestor of Group 1 at 1988 (1979–1995) and Group 2 at 1984 (1968–1998). Our evolutionary rate estimate is lower than some estimates for *M. tuberculosis* in humans, (95% CIs: 0.3–0.7 substitutions genome^−1^ year^−1^ (Roetzer et al., 2013, Walker et al., 2012), whereas other studies reported similar rates (0.13–0.41; Bryant et al., 2013b). While differences in pathogen life history, such as disease latency (Colangeli et al., 2014) or non-replicating persistence of bacteria in the environment (Courtenay et al., 2006, Maddock, 1933, Young et al., 2005), might contribute to rate varition among different myobacteria, further studies will be required to verify this.

### 3.5 Comparing genetic and epidemiological relationships between breakdowns

A Mantel test showed a significant, though weak, association between the genetic and spatial distances of Group 1 breakdowns (*p* = 0.014, Spearman's rank coefficient = 0.20; Fig. S4), as did multiple regression on matrices (*p* = 0.034, *R*^2^ = 0.031).

**Fig. 3:**
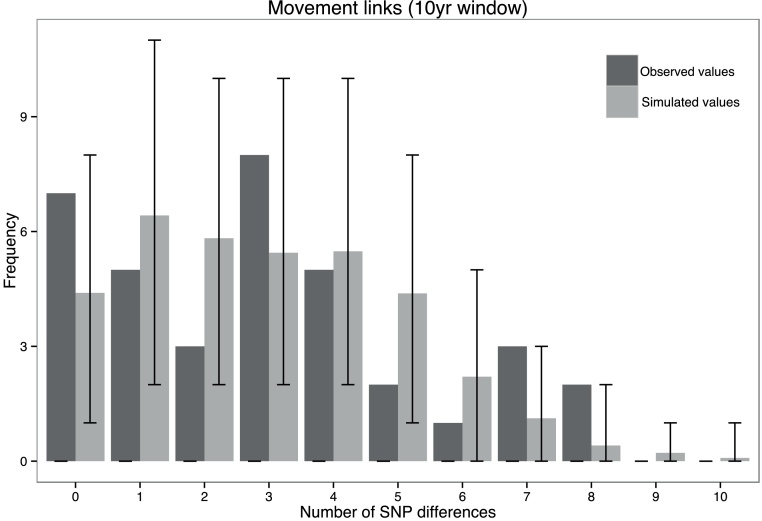
Observed number of SNP differences between outbreaks linked by movements of cattle within a 10-year timeframe (dark grey), and expected SNP differences from 10^4^ simulations of the null hypothesis of no association between presence of a link and genetic similarity (light grey). Bars show the intervals containing 95% of the results from the null simulations.

**Fig. 4:**
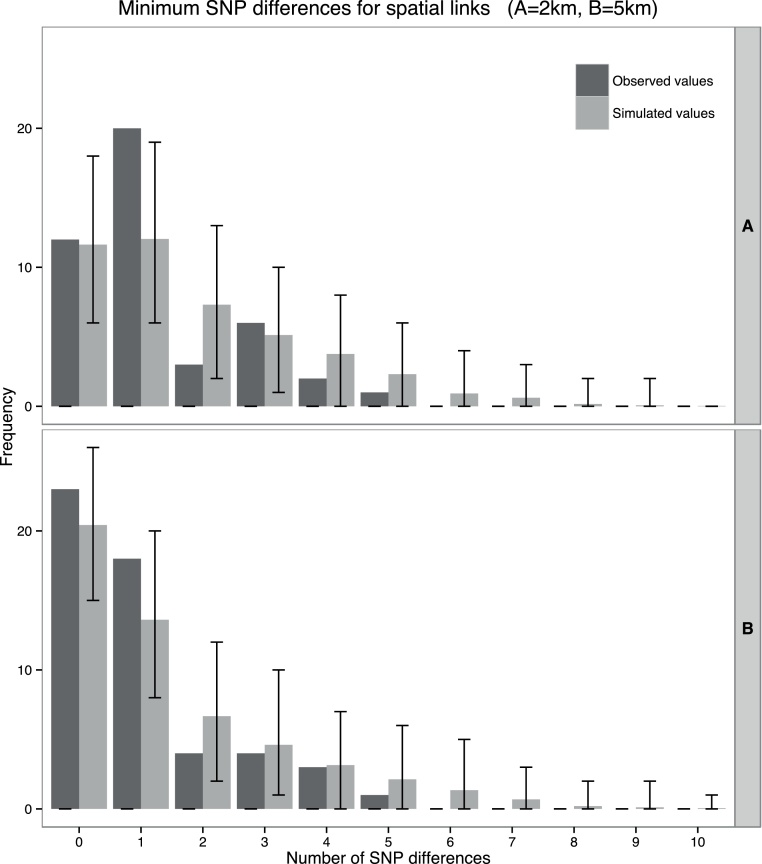
Observed number of SNP differences between outbreaks linked by spatial proximity (dark grey), and expected SNP differences from 10^4^ simulations of the null hypothesis of no association between presence of a link and genetic similarity (light grey). Bars give the intervals containing 95% of the results from the null simulations. A. shows results for spatial proximity of 2 km and B. shows results for 5 km.

The minimum SNP differences observed between pairs of sequenced Group 1 breakdowns linked by direct recorded cattle movements (Fig. 3, mean 1.29 SNP differences, range 0–11 SNPs) and by spatial proximity of 5 km (Fig. 4B, mean 0.49 SNPs, range 0–4 SNPs) were not significantly different from expectations under the null hypothesis of no association (*p*-values of 0.350 and 0.338 respectively), whereas the association showed borderline significance for spatial proximities of 2 km (Fig. 4A: mean 0.57 SNPs, range 0–11 SNPs, *p* = 0.048). These analyses were also conducted on a subset of the Group 1 data comprising isolates occurring from 2009 onwards (i.e., after sampling intensity was increased to include all infected cattle in a bTB breakdown), to check for an effect of temporal differences in sampling intensity, and these also gave non-significant results (Fig. S5).

Four of the Group 1 VNTR-10 breakdowns from which isolates were sequenced for this study showed no apparent epidemiological links to any other breakdown; neither through direct recorded movements nor spatial proximity up to 5 km (Fig. S6). One of the Group 2 VNTR-10 breakdown had no apparent epidemiological links to other VNTR-10 isolates, while the other two Group 2 breakdowns did show epidemiological links, but are only comparatively distantly related to the linked breakdowns (>10 SNP differences).

Data from repeat breakdowns of VNTR-10 within the same herd were available for 11 herds and 27 breakdowns. For nine of these breakdowns, the later breakdown was caused by an isolate closely related to the preceding breakdown (less than 3 SNP differences), indicating the possibility of local persistence even after a cattle herd has been declared free of bTB.

### 3.6 Application of phylogeographic tools

Comparison of different phylogeographic models of bacterial dispersal showed statistical support for a heterogeneous model of spatial diffusion allowing different rates of spread among phylogenetic branches (log MLEs: −1518 for the relaxed model and −1605 for the homogeneous model). This model further provides information for each phylogenetic branch about the estimated distance travelled over the time period represented by its length. Based on the MCC tree for Group 1 (terminal branches only), the estimated mean diffusion rate was comparatively low at 2 km/year, but with higher rates up to 30 km/year seen rarely (Fig. S7). The majority of branches underlying these rates involve distances of <5 km travelled over less than 5 years (Fig. S8).

In 95 out of 100 simulations of homogeneous spatial diffusion along the time-stamped VNTR-10 phylogeny, the originally-specified diffusion rate could be recovered in BEAST, in that the originally specified value fell within the estimated 95% HPD (Fig. S9). This suggests that, despite low levels of genetic divergence, our data contain sufficient phylogenetic signal for meaningful phylogeographic inference.

## 4 Discussion

Applying WGS to an intensively sampled molecular type of *M. bovis* in cattle allowed us to explore the potential of this approach to assess the role of cattle movements or spatial proximity in transmission, and to quantify bacterial dispersal across the landscape. Our findings not only demonstrate the potential of WGS as a tool for epidemiological investigation of bTB, but also clearly expose certain limitations.

### 4.1 Differential sampling intensity between VNTR-10 clades and switching of VNTR-type

As expected for a well-sampled population of slowly evolving bacteria, the majority of VNTR-10 isolates (specifically: Group 1) were genetically highly similar, often identical, and included most of the recent ancestral sequence types that can be inferred from the VNTR-10 phylogeny. In contrast, we found a small number of VNTR-10 samples (Group 2, Fig. 1) that were phylogenetically distinct from the Group 1 samples and showed higher pairwise genetic diversity. Sampling and sequencing effort was even across all VNTR-10-typed isolates from cattle, so the finding of a rare group with more divergent isolates was surprising and suggests that our sampling of Group 2 isolates was less complete compared to Group 1. Observing a much smaller proportion of the overall bacterial diversity might have suggested that this lineage was maintained in a host population largely missed by our sampling, such as a non-cattle reservoir host. However, the sequence data presented here from the closely related VNTR-1 strain indicates that the apparent under-sampling in the Group 2 was caused by switching of VNTR phenotype within this lineage. The placement of VNTR-1 isolates C and D implies that the emergence of VNTR-10 from VNTR-1 occurred independently for the Group 1 clade, whereas the relationship between VNTR-1 samples A and B, and VNTR-10 samples in Group 2 (samples 1–3) suggests that in Group 2 bacteria the VNTR-type has switched VNTR-type multiple times between VNTRs-1 and -10 (Fig. 1). Because our sampling strategy was reliant on VNTR-typing, and focussed on VNTR-10, lineages that had changed to a different type were almost certain to be missed. Similar evidence for type switching from whole genome data has also recently emerged for human TB (Bryant et al., 2013a, Walker et al., 2012) and in other mycobacteria (Ahlstrom et al., 2015).

Given that we have sequenced so few VNTR-1 isolates, the finding that all of them cluster within the VNTR-10 samples is significant, however it is difficult to say whether the Group 2 lineage is genuinely more prone to VNTR-switching than Group 1. Although studies have found only limited phenotypic differences between different molecular types of *M. bovis* (Wright et al., 2013a, Wright et al., 2013b), it is possible that a difference between the two groups of VNTR-10 isolates, in terms of their propensity for VNTR-switching, may be related to functional genetic differences between them. Two out of the four SNPs differentiating the clades show non-synonymous changes in annotated regions, one in the *S*th *A* gene and one in a region coding for an unknown hypothetical protein. Additionally, the MV2163B locus which differentiates VNTRs-1 and -10 (Table S2) is known to occur within the open reading frame of PPE gene Rv1917c, one of a family of proteins thought to play a role in antigenic variation (Skuce et al., 2002). Therefore a difference in VNTR-switching between clades could also be a response to selective pressures acting on this VNTR locus. However, MV2163B does not show any greater diversity of tandem repeat variation than other NI VNTR-loci, and if anything appears slightly less prone to variation than the others (Skuce et al., 2005).

### 4.2 Comparing genetic and epidemiological relationships between breakdowns

An analysis of the association between close spatial proximity among breakdowns (2 km and 5 km) and genetic similarity gave only marginally significant results (*p* = 0.048) for distances of under 2 km, and showed no significance for distances of under 5 km (Fig. 4). A similar evaluation of the correlation between direct recorded movements of cattle between breakdowns and genetic similarity also showed no significant associations (Fig. 3).

In contrast to findings for more rapidly evolving bacteria (Eyre et al., 2013), our results suggest that the use of pairwise distances to track transmission and to assess the relative roles of potential transmission mechanisms lacks power for *M. bovis*. The lack of power seen here is likely to be due to the low level of genetic signal in this slowly evolving pathogen as well as the variable, often prolonged, duration of infection within an animal. As a consequence, the genetic distance between breakdowns will be difficult to predict even for breakdowns linked by direct transmission of infection. Adding to this genetic uncertainty, over distances of 2 and 5 km the discrepancy between the registered location of a herd and the actual location of the cattle will be brought to the fore (especially in NI where use of rented pasture is common and level of farm fragmentation is high (Abernethy et al., 2006)), and recorded movements assessed here do not include indirect movements between herds which may also play a role in transmission.

On a broader scale however, the significant, though weak, correlation between genetic and spatial distances within Group 1 breakdowns demonstrated by the Mantel test and multiple regression on matrices indicates that spatially localised mechanisms are likely involved in the transmission of this lineage. This is consistent with earlier findings (Biek et al., 2012), as well as the large-scale patterns of spatial expansion of the bTB-endemic areas in the southwest of Britain (Brunton et al., 2015).

### 4.3 Application of phylogeographic tools

Despite the limitations described above, phylogenetic data for *M. bovis* can provide insight into the pattern and process of spatial spread. Encouragingly, even taking into account the estimation uncertainties and the low evolutionary rate discussed above, our simulations demonstrate that, genome-wide variation of *M. bovis* contains sufficient information to support meaningful Bayesian phylogeographic analyses over the temporal and spatial scales covered by our data. Based on such an approach, a heterogeneous model of spatial spread fitted the WGS data significantly better than a model assuming a homogeneous diffusion process, implying that the VNTR-10 group has spread across the landscape at a variable rate. Such a pattern might indicate that transmission is underpinned by multiple mechanisms, each associated with a different diffusion process and rate, or it may suggest that transmission is largely driven by a single mechanism involving a variable rate of spatial spread (for example, human-mediated movements of cattle between herds). The low mean spatial diffusion rate of VNTR-10 of 2 km/year fits the observation of strong spatial clustering characteristic of *M. bovis* in the UK, seen over different scales and typing methods (Skuce et al., 2010, Smith et al., 2006).

### 4.4 Implications for bTB management

Our finding that homoplasy due to VNTR-switching may be common in bTB is significant from an applied point of view since it could confound the epidemiological distinctions made between closely related VNTR-types. However, VNTR-typing in NI is used in conjunction with spoligotyping, and the VNTR loci used have been chosen for optimal discrimination within the NI *M. bovis* population (Skuce et al., 2005), both of which are expected to reduce the impact of VNTR-type homoplasies (Reyes et al., 2012). Of the ten most common VNTR-types in NI (accounting for 85% of all VNTR-typed isolates (Skuce et al., 2010)), only one pair is separated by less than two VNTR tandem repeat differences while sharing the same spoligotype. VNTR-switching is therefore expected to have limited impact on the routine application of VNTR-typing for bTB in NI. Additionally, the VNTR-typing of all culture positive animals in a breakdown (currently standard in NI) will facilitate early detection of VNTR-switching events should they occur.

This study also suggests that it may be possible in principle to use WGS to identify under-sampled populations in *M. bovis*, in this case due to switching of VNTR-type between VNTR-10 and the closely related VNTR-1*.* However, whether WGS will provide sufficient resolution to characterise the involvement of under-sampled wildlife reservoirs for *M. bovis* is unclear, and inferences will also be affected by the rate at which transmission occurs between the two host populations (Kao et al., 2014).

Despite the exceptional quality of epidemiological data available for *M. bovis* in NI and the high intensity of sampling, we found that four Group 1 VNTR-10 breakdowns in this study showed no links to other VNTR-10 breakdowns, neither through direct recorded movements within 10 years of the breakdown nor through spatial proximity of 5 km or less. Although in Group 2 samples such a finding is likely due to the under-sampling of this lineage due to VNTR-switching, the presence of “unlinked” breakdowns in Group 1 is more surprising, suggesting that the epidemiological links assessed here do not cover all the routes through which infection spreads.

We suggest due to its slow evolutionary rate, some limitations will always be inherent in the application of WGS to *M. bovis* epidemiology and accordingly care must be taken in interpreting results: certain analyses will always remain problematic, for example, unambiguous determination of the underlying transmission tree, “who infected whom” (Didelot et al., 2014, Köser et al., 2012). However, we show here that other approaches, such as Bayesian phylogeographic techniques to explore the spatial spread of disease, appear feasible for this pathogen.

## 5 Conclusion

Despite a rate of evolution amongst the lowest recorded to date among bacteria, the genomic data presented show a substantial improvement in genetic resolution over previous methods of genetic typing. While WGS data have considerable potential to enhance both our in-depth understanding of bTB epidemiology as well as routine bTB surveillance, the slow evolutionary rate of *M. bovis* does impose a limit to this potential, as has been noted in human tuberculosis (e.g., Didelot et al., 2014, Köser et al., 2012). For the future, we suggest that continued advances in mathematical models integrating epidemiological and genetic information will allow a more confident resolution of the factors involved in the spread of bovine tuberculosis, giving a better understanding of the interplay between epidemiological and genetic factors for this important and troubling pathogen.

## Funding

The Wellcome Senior Research Fellowship acknowledged as a funder has a Wellcome Reference 081696/Z/06/Z.
