# Supplementary material for: Use of bacterial whole-genome sequencing to investigate local persistence and spread in bovine tuberculosis
Source: Epidemics. 2016 Mar;14:26–35. doi: 10.1016/j.epidem.2015.08.003 (PMC4773590; doi:10.1016/j.epidem.2015.08.003)
Supplement: Supplementary file 1 [file mmc1.pdf]

Use of bacterial whole-genome sequencing to investigate local persistence and spread in bovine tuberculosis: Supplemental Information

STATISTICAL COMPARISON OF VNTR-10 WITH OTHER NI VNTR-TYPES

VNTR-10 is a recently emerged NI VNTR-type, located predominantly within the Newtownards area of NI (178/195 VNTR-10 infections recorded between 1996-2011 were from Newtownards). To confirm whether the VNTR-10 type is representative of other NI VNTR-types, we compared eight known risk factors for bTB infection (Skuce et al., 2012) in both VNTR-10 breakdowns and breakdowns attributable to other *M. bovis* strains (Table 1; a breakdown is attributed to the first VNTR-type isolated from that breakdown). Records of 10,751 breakdowns attributable to other strains that began between 2003 and 2011 were identified were linked with herd level risk factors and compared to 94 VNTR-10 breakdowns (1996-2011: all VNTR-10 outbreaks were retained for VNTR-10 to maximise the relatively small number of breakdowns available) using R v2.14 (R Core Team, 2014). Two comparisons were made, first using all herd breakdowns in NI as the comparator population, and secondly at a local scale, using only breakdowns in the Newtownards area (results shown in Table 1). While there were several significant differences in risk factors between VNTR-10 breakdowns and those attributed to other strains of *M. bovis* across NI, all except one of these factors were non-significant in the comparison using Newtownards strains only ($p>0.05$). Only the number of neighbouring cattle herds for VNTR-10 breakdowns was significantly lower for bTB breakdowns attributable to other strains in both the local area ($p=0.004$) and across NI as a whole ($p<0.001$).

It is possible that the lower numbers for the Newtownards-only comparison may be acting to reduce statistical power. However, if this does indicate genuine similarities between VNTR-10 and other Newtownards VNTR-types, it implies that the differences seen at the whole NI level result from differences between Newtownards and other areas rather than factors specific to the VNTR-10 molecular type. Previous studies have also shown no significant effect of *M. bovis* strain type on skin test result or outbreak size (Wright et al., 2013a, 2013b), and although phenotypic differences are noted between different lineages of *M. tuberculosis*, this could be due to the higher levels of diversity in this species (Coscolla and Gagneux, 2014; Rose et al., 2013). Taken together this suggests that VNTR-10 should be representative of bTB in NI more generally and should also be broadly informative with respect to epidemiological factors.

BIOINFORMATICS AND SNP CALLING

Paired end raw reads from 31 *M. bovis* isolates sequenced for the previous study (Biek et al., 2012; Batch 1) were combined with 116 *M. bovis* samples sequenced separately as paired ends for this study using an Illumina IIx ("Batch 2") and 3 *M. bovis* VNTR-1 isolates (samples B-D, "Batch 3"), also paired ends, sequenced using an Illumina MiSeq. These isolates were processed using the following workflow.

Reads were trimmed based on quality using ConDeTri (Smeds and Kunstner, 2011), then BWA v0.7.5a (Li and Durbin, 2009) was used at the default settings to assemble the reads to

the *M. bovis* reference genome AF2122/97 (GenBank accession number BX248333; Garnier et al., 2003). SAMtools mpileup and bcftools were then applied to identify variant sites within the assembly (Li et al., 2009).

Code was written in Python to filter the raw variants identified above according to: total depth of coverage; depth of high quality coverage (according to the SAMtools output vcf file) on the forward and reverse strands; mapping quality; and percentage of high quality bases that give the dominant allele (*M. bovis* is a slowly mutating, haploid organism, and the sequenced isolates are grown from pure cultures, therefore true variant sites are expected to show minimal heterozygosity).

The filter code was run using two settings, giving a strict filter and a relaxed filter (Table S3). The percentage of high quality bases giving the dominant allele (homozygosity) was kept at 95% for both filters, as lower values appeared to generate homoplasy (see below). The *M. bovis* population as a whole shows very little genetic diversity, especially in Britain and Ireland (Smith et al., 2006), and these samples represent only one closely-related strain within that limited diversity. Therefore, across the whole genome, true biological variation is expected to be rare and any variant identified has a high chance of being the result of sequencing error, and so for our purposes SNP calling needed to be as stringent as possible. Initially the strict filter, with high filter thresholds, was run to identify sites across the genome where at least one sample contained a high quality variant. Once these sites containing high quality SNPs had been identified, variants at these sites in other samples have an increased likelihood of being true biological variants, and accordingly, the more relaxed filter with lower thresholds was then used to determine whether each of the samples pass or fail at each high-quality variant site, and if pass, whether reference or SNP.

After the filtering code was run and sites containing a high quality SNP in at least one isolate were identified, these sites were further filtered and removed if:

- They clustered together with another variant site less than 200bp apart (if mutations were distributed evenly through the genome, we would expect a SNP every ~14000bp). Clustering of variants may suggest an area of the genome prone to sequencing or mapping error, or alternatively, clustered SNPs might indicate areas of recombination or selection. However, either of these explanations would confound our downstream analysis and therefore these SNPs were removed from the final dataset.
- They were in a repeat region of the genome, as identified previously by artificially fragmenting the reference genome, and using BLAST to identify regions receiving more than the expected number of BLAST hits (Biek et al., 2012). Sites identified using these criteria are given as a supplementary text file.
- More than 10% of samples failed the relaxed filter criteria at that site

The pass-reference/pass-variant/fail information given by the relaxed filter was then used to generate sequences of concatenated variant sites for each of the samples (sites failing the filter were denoted 'N').

The ideal variant filter thresholds are those that remove all error variants while leaving as many of the true variant sites as possible. Given the clonality of *M. bovis* bacteria (Hershberg et al., 2008; Pepperell et al., 2013; Wirth et al., 2008), all true SNP sites should support one phylogenetic tree, and therefore any homoplasies would indicate that error variants are present in the data. Using this, the filter criteria were sequentially lowered and the optimum filter thresholds (Table S3) were determined to be those just high enough to prevent homoplasies.

Batch 1 samples generated considerably more reads, and therefore a higher depth of coverage, than Batches 2 and 3. For this reason, the total depth and the high quality depth filters were increased for Batch 1 samples, to the point where they generated on average the same number of variant sites per sample as the filter criteria used for Batches 2 and 3.

Post assembly, the mean depth of coverage of the *M. bovis* genome was 108 reads for Batch 1 isolates, 53 reads for Batch 2 isolates and 45 for Batch 3, with more than 97% of the genome covered by at least one high quality base in all batches. 335 sites that varied from the reference genome were identified by the strict filter criteria, and 290 of these survived all the filtering steps and were used for downstream analyses; 41 of these sites were informative to the VNTR1/10 group (i.e. shared by two or more isolates within this group).

BAYESIAN PHYLOGENETIC PARAMETERS

BEAST analyses were run in BEAST v1.7.4, using the Jukes Cantor model of nucleotide substitution and the Bayesian skyline model as demographic coalescent prior (Drummond et al., 2005). The prior for the molecular clock rate was given as a normal distribution with the mean corresponding to a rate estimate for human tuberculosis (0.5 SNPs per genome per year; Walker et al., 2012), but with a wider standard deviation (>10 SNPs per genome per year) to account for the fact that we are investigating a different bacterial system. Convergence was determined as effective sample sizes (ESS) >200 for all parameters, as determined in Tracer (v1.5, http://beast.bio.ed.ac.uk/software/tracer/). Log marginal likelihood estimates (MLEs) were calculated to assess model fit using path-sampling and stepping-stone sampling (Baele et al., 2012), run twice to assess MLE convergence.

In order to ascertain the correct clock model to use for the Bayesian phylogeographic analysis, BEAST was used to fit and compare three different molecular clock models: a strict clock model and two relaxed clock models allowing evolutionary rates to differ among branches, with rates drawn from log-normal or exponential distributions (Drummond et al., 2006). The analyses were run for 500 million iterations of the MCMC chain, resulting in convergence.

Log marginal likelihood estimates for the strict molecular clock and the relaxed lognormal clock models were almost identical (-1504.29 and -1504.85 respectively), and both models were slightly preferred over the relaxed exponential clock (log marginal likelihood estimate: 1508.22). These results indicate that a strict molecular clock model is appropriate for these data, and therefore the strict model was used in all subsequent BEAST analyses, in combination with the other parameter values specified above

To assess whether the priors chosen for BEAST analyses shaped the results for these analyses, we ran BEAST for 10^{10} MCMC iterations, using the same settings described above but sampling from the prior distributions only. However, these runs failed to converge (combined ESS value of 31 for the tree posterior).

REFERENCES

Baele, G., Lemey, P., Bedford, T., Rambaut, A., Suchard, M.A., Alekseyenko, A. V, 2012. Improving the accuracy of demographic and molecular clock model comparison while accommodating phylogenetic uncertainty. Mol. Biol. Evol. 29, 2157–2167. doi:10.1093/molbev/mss084

Biek, R., O'Hare, A., Wright, D., Mallon, T., McCormick, C., Orton, R.J., McDowell, S., Trewby, H., Skuce, R.A., Kao, R.R., 2012. Whole genome sequencing reveals local transmission patterns of Mycobacterium bovis in sympatric cattle and badger populations. PLoS Pathog. 8, e1003008. doi:10.1371/journal.ppat.1003008

Drummond, A.J., Ho, S.Y.W., Phillips, M.J., Rambaut, A., 2006. Relaxed phylogenetics and dating with confidence. PLoS Biol. 4, e88. doi:10.1371/journal.pbio.0040088

Drummond, A.J., Rambaut, A., Shapiro, B., Pybus, O.G., 2005. Bayesian coalescent inference of past population dynamics from molecular sequences. Mol. Biol. Evol. 22, 1185–1192. doi:10.1093/molbev/msi103

Garnier, T., Eiglmeier, K., Camus, J.C., Medina, N., Mansoor, H., Pryor, M., Duthoy, S., Grondin, S., Lacroix, C., Monsempe, C., Simon, S., Harris, B., Atkin, R., Doggett, J., Mayes, R., Keating, L., Wheeler, P.R., Parkhill, J., Barrell, B.G., Cole, S.T., Gordon, S. V, Hewinson, R.G., 2003. The complete genome sequence of Mycobacterium bovis. Proc. Natl. Acad. Sci. 100, 7877–7882. doi:10.1128/genomeA.00069-13

Hershberg, R., Lipatov, M., Small, P.M., Sheffer, H., Niemann, S., Homolka, S., Roach, J.C., Kremer, K., Petrov, D.A., Feldman, M.W., Gagneux, S., 2008. High functional diversity in Mycobacterium tuberculosis driven by genetic drift and human demography. PLoS Biol. 6, e311. doi:10.1371/journal.pbio.0060311

Li, H., Durbin, R., 2009. Fast and accurate short read alignment with Burrows-Wheeler transform. Bioinformatics 25, 1754–1760. doi:10.1093/bioinformatics/btp324

Li, H., Handsaker, B., Wysoker, A., Fennell, T., Ruan, J., Homer, N., Marth, G., Abecasis, G., Durbin, R., 2009. The Sequence Alignment/Map format and SAMtools. Bioinformatics 25, 2078–2079. doi:10.1093/bioinformatics/btp352

Pepperell, C.S., Casto, A.M., Kitchen, A., Granka, J.M., Cornejo, O.E., Holmes, E.C., Birren, B., Galagan, J., Feldman, M.W., 2013. The role of selection in shaping diversity of natural M. tuberculosis populations. PLoS Pathog. 9, e1003543. doi:10.1371/journal.ppat.1003543

Skuce, R.A., McCorry, T.P., McCarroll, J.F., Roring, S.M.M., Scott, A.N., Brittain, D., Hughes, S.L., Hewinson, R.G., Neill, S.D., 2002. Discrimination of Mycobacterium tuberculosis complex bacteria using novel VNTR-PCR targets. Microbiology 148, 519–528.

Skuce, R.A., McDowell, S.W., Mallon, T.R., Luke, B., Breadon, E.L., Lagan, P.L., McCormick, C.M., McBride, S.H., Pollock, J.M., 2005. Discrimination of isolates of Mycobacterium bovis in Northern Ireland on the basis of variable numbers of tandem repeats (VNTRs). Vet. Rec. 157, 501–504.

Smeds, L., Kunstner, A., 2011. ConDeTri - A content dependent read trimmer for Illumina data. PLoS One 6, s26314. doi:10.1371/journal.pone.0026314

Smith, N.H., Gordon, S. V, de la Rua-Domenech, R., Clifton-Hadley, R.S., Hewinson, R.G., 2006. Bottlenecks and broomsticks: the molecular evolution of Mycobacterium bovis. Nat. Rev. Microbiol. 4, 670–681. doi:10.1038/nrmicro1472

Walker, T.M., Ip, C.L.C., Harrell, R.H., Evans, J.T., Kapatai, G., Dedicoat, M.J., Eyre, D.W., Wilson, D.J., Hawkey, P.M., Crook, D.W., Parkhill, J., Harris, D., Walker, A.S., Bowden, R., Monk, P., Smith, E.G., Peto, T.E.A., 2012. Whole-genome sequencing to delineate Mycobacterium tuberculosis outbreaks: a retrospective observational study. Lancet Infect. Dis. 3099. doi:10.1016/S1473-3099(12)70277-3

Wirth, T., Hildebrand, F., Allix-Béguec, C., Wölbeling, F., Kubica, T., Kremer, K., van Soolingen, D., Rüsch-Gerdes, S., Locht, C., Brisse, S., Meyer, A., Supply, P., Niemann, S., 2008. Origin, spread and demography of the Mycobacterium tuberculosis complex. PLoS Pathog. 4, e1000160. doi:10.1371/journal.ppat.1000160

Wright, D.M., Allen, A.R., Mallon, T.R., McDowell, S.W.J., Bishop, S.C., Glass, E.J., Bermingham, M.L., Woolliams, J.A., Skuce, R.A., 2013a. Field-isolated genotypes of Mycobacterium bovis vary in virulence and influence case pathology but do not affect outbreak size. PLoS One 8, e74503. doi:10.1371/journal.pone.0074503

Wright, D.M., Allen, A.R., Mallon, T.R., McDowell, S.W.J., Bishop, S.C., Glass, E.J., Bermingham, M.L., Woolliams, J.A., Skuce, R.A., 2013b. Detectability of bovine TB using the tuberculin skin test does not vary significantly according to pathogen genotype within Northern Ireland. Infect. Eenetics Evol. 19, 15–22. doi:10.1016/j.meegid.2013.05.011
