# Supplementary material for: Use of bacterial whole-genome sequencing to investigate local persistence and spread in bovine tuberculosis
Source: Epidemics. 2016 Mar;14:26–35. doi: 10.1016/j.epidem.2015.08.003 (PMC4773590; doi:10.1016/j.epidem.2015.08.003)
Supplement: Supplementary file 2 [file mmc2.pdf]

Figure S1: Minimum spanning tree generated using the R package Pegas [1] showing relationships between Northern Irish VNTR-types belonging to spoligotype SB0140 (the most common spoligotype in NI). Circles are scaled to be proportionate to the number of times the VNTR-type was recorded.

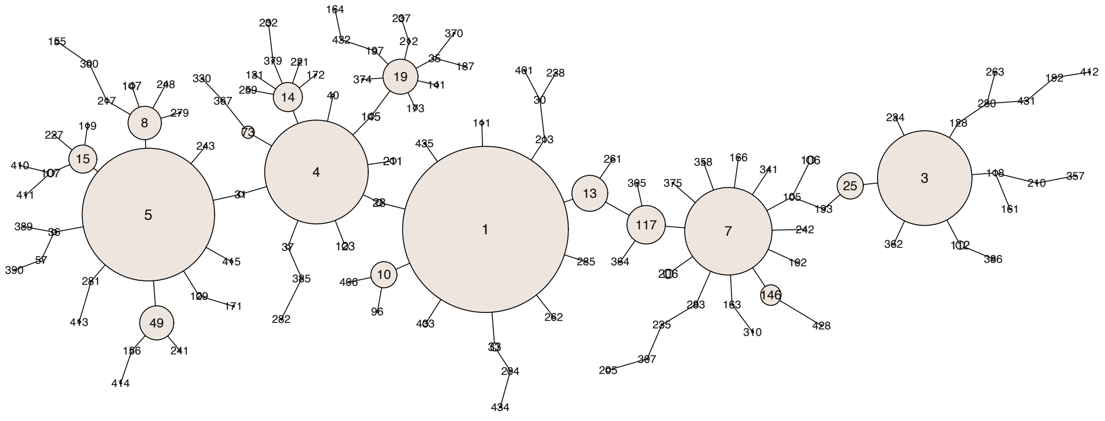

Figure S2: (next page) ML phylogeny of all isolates described in the study, rooted on the *M. bovis* reference sequence AF2122−97 and the VNTR-4 isolate (both shown in the figure). Phylogeny generated in PhyML v3.0 [2] using the Jukes-Cantor model of nucleotide substitution. Isolates included in the downstream analyses are shown in red bold font. Tip labels give epidemiological information in the following order: sample reference (corresponding to Table S1), anonymised herd ID, anonymised breakdown ID, year of sampling.

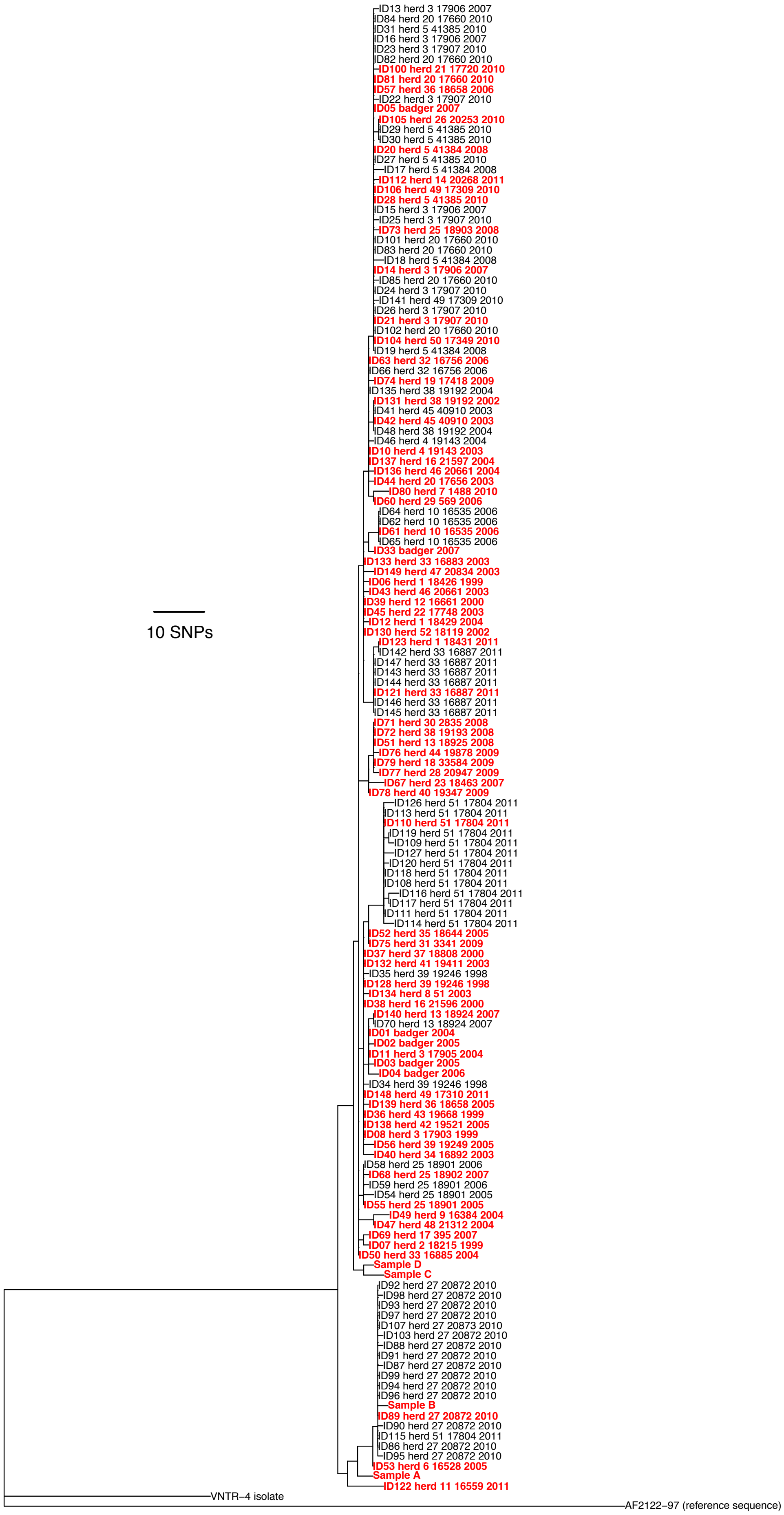

ID13 herd 3 17906 2007
ID84 herd 20 17660 2010
ID31 herd 5 41385 2010
ID16 herd 3 17906 2007
ID23 herd 3 17907 2010
ID82 herd 20 17660 2010
ID100 herd 21 17720 2010
ID81 herd 20 17660 2010
ID57 herd 36 18658 2006
ID22 herd 3 17907 2010
ID05 badger 2007
ID105 herd 26 20253 2010
ID29 herd 5 41385 2010
ID30 herd 5 41385 2010
ID20 herd 5 41384 2008
ID27 herd 5 41385 2010
ID17 herd 5 41384 2008
ID112 herd 14 20268 2011
ID106 herd 49 17309 2010
ID28 herd 5 41385 2010
ID15 herd 3 17906 2007
ID25 herd 3 17907 2010
ID73 herd 25 18903 2008
ID101 herd 20 17660 2010
ID83 herd 20 17660 2010
ID18 herd 5 41384 2008
ID14 herd 3 17906 2007
ID85 herd 20 17660 2010
ID24 herd 3 17907 2010
ID141 herd 49 17309 2010
ID26 herd 3 17907 2010
ID21 herd 3 17907 2010
ID102 herd 20 17660 2010
ID104 herd 50 17349 2010
ID19 herd 5 41384 2008
ID63 herd 32 16756 2006
ID66 herd 32 16756 2006
ID74 herd 19 17418 2009
ID135 herd 38 19192 2004
ID131 herd 38 19192 2002
ID41 herd 45 40910 2003
ID42 herd 45 40910 2003
ID48 herd 38 19192 2004
ID46 herd 4 19143 2004
ID10 herd 4 19143 2003
ID137 herd 16 21597 2004
ID136 herd 46 20661 2004
ID44 herd 20 17656 2003
ID80 herd 7 1488 2010
ID60 herd 29 569 2006
ID64 herd 10 16535 2006
ID62 herd 10 16535 2006
ID61 herd 10 16535 2006
ID65 herd 10 16535 2006
ID33 badger 2007
ID133 herd 33 16883 2003
ID149 herd 47 20834 2003
ID06 herd 1 18426 1999
ID43 herd 46 20661 2003
ID39 herd 12 16661 2000
ID45 herd 22 17748 2003
ID12 herd 1 18429 2004
ID130 herd 52 18119 2002
ID123 herd 1 18431 2011
ID142 herd 33 16887 2011
ID147 herd 33 16887 2011
ID143 herd 33 16887 2011
ID144 herd 33 16887 2011
ID121 herd 33 16887 2011
ID146 herd 33 16887 2011
ID145 herd 33 16887 2011
ID71 herd 30 2835 2008
ID72 herd 38 19193 2008
ID51 herd 13 18925 2008
ID76 herd 44 19878 2009
ID79 herd 18 33584 2009
ID77 herd 28 20947 2009
ID67 herd 23 18463 2007
ID78 herd 40 19347 2009
ID126 herd 51 17804 2011
ID113 herd 51 17804 2011
ID110 herd 51 17804 2011
ID119 herd 51 17804 2011
ID109 herd 51 17804 2011
ID127 herd 51 17804 2011
ID120 herd 51 17804 2011
ID118 herd 51 17804 2011
ID108 herd 51 17804 2011
ID116 herd 51 17804 2011
ID117 herd 51 17804 2011
ID111 herd 51 17804 2011
ID114 herd 51 17804 2011
ID52 herd 35 18644 2005
ID75 herd 31 3341 2009
ID37 herd 37 18808 2000
ID132 herd 41 19411 2003
ID35 herd 39 19246 1998
ID128 herd 39 19246 1998
ID134 herd 8 51 2003
ID38 herd 16 21596 2000
ID140 herd 13 18924 2007
ID70 herd 13 18924 2007
ID01 badger 2004
ID02 badger 2005
ID11 herd 3 17905 2004
ID03 badger 2005
ID04 badger 2006
ID34 herd 39 19246 1998
ID148 herd 49 17310 2011
ID139 herd 36 18658 2005
ID36 herd 43 19668 1999
ID138 herd 42 19521 2005
ID08 herd 3 17903 1999
ID56 herd 39 19249 2005
ID40 herd 34 16892 2003
ID58 herd 25 18901 2006
ID68 herd 25 18902 2007
ID59 herd 25 18901 2006
ID54 herd 25 18901 2005
ID55 herd 25 18901 2005
ID49 herd 9 16384 2004
ID47 herd 48 21312 2004
ID69 herd 17 395 2007
ID07 herd 2 18215 1999
ID50 herd 33 16885 2004
Sample D
Sample C
ID92 herd 27 20872 2010
ID98 herd 27 20872 2010
ID93 herd 27 20872 2010
ID97 herd 27 20872 2010
ID107 herd 27 20873 2010
ID103 herd 27 20872 2010
ID88 herd 27 20872 2010
ID91 herd 27 20872 2010
ID87 herd 27 20872 2010
ID99 herd 27 20872 2010
ID94 herd 27 20872 2010
ID96 herd 27 20872 2010
Sample B
ID89 herd 27 20872 2010
ID90 herd 27 20872 2010
ID115 herd 51 17804 2011
ID86 herd 27 20872 2010
ID95 herd 27 20872 2010
ID53 herd 6 16528 2005
Sample A
ID122 herd 11 16559 2011
VNTR−4 isolate
AF2122−97 (reference sequence)
10 SNPs

Figure S3: True pairwise genetic distances for Group 2 VNTR-10 sequences, and pairwise distances generated from 1000 subsamples of Group 1 sequences, with and without weighting for sampling year (A, B and C, respectively)

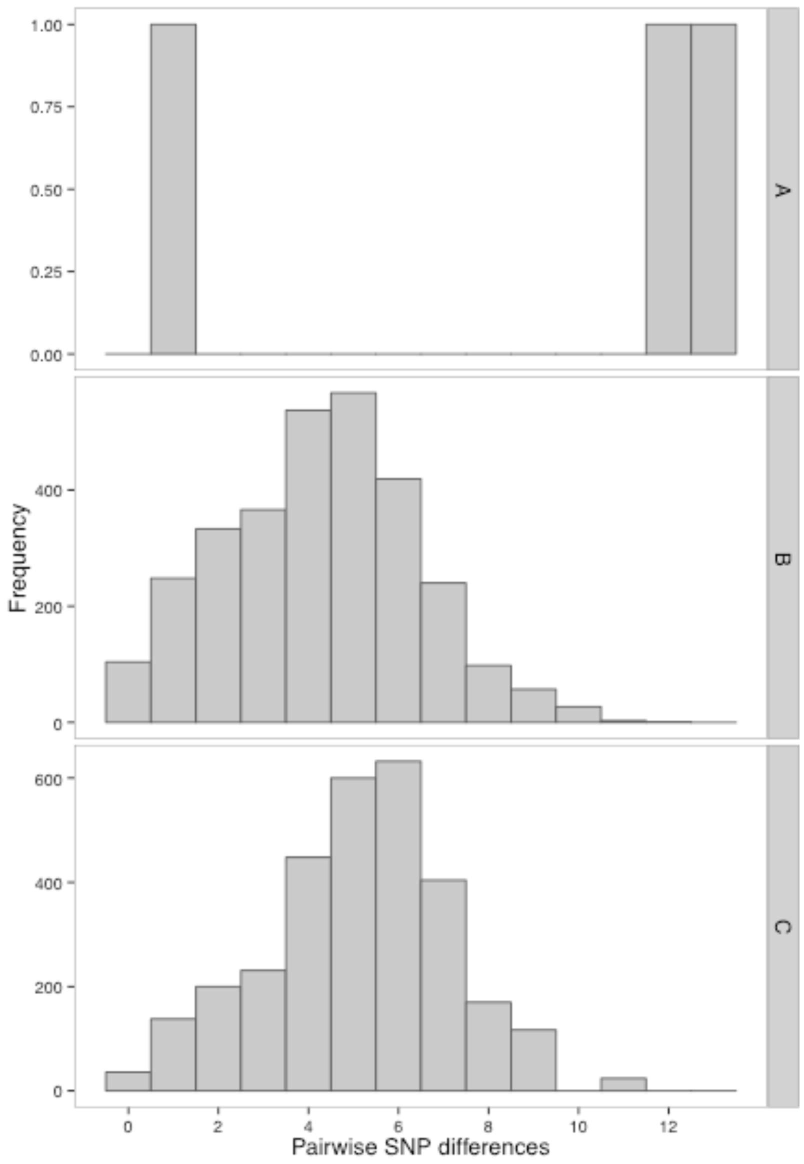

Figure S4: Comparison of pairwise genetic and spatial distances between all Group 1 herd breakdowns. Pairs of breakdowns linked by direct recorded movements of cattle within a 10 year window are shown in red, all others are shown in grey.

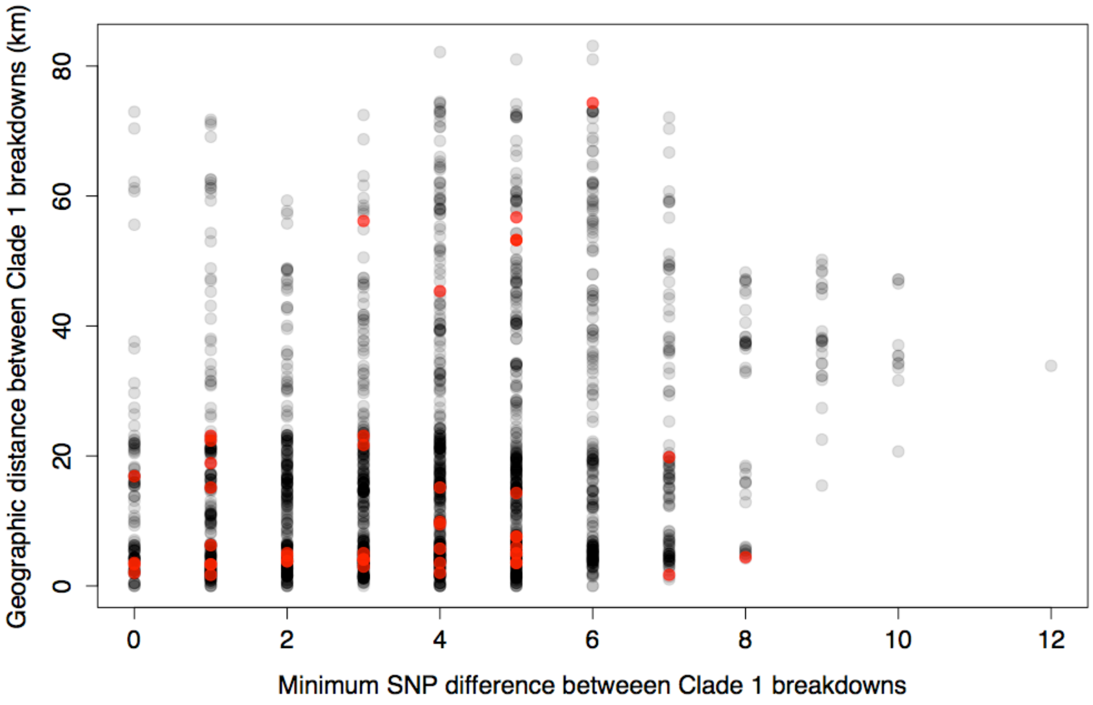

Figure S5: Observed number of SNP differences between outbreaks occurring from 2009 onwards, linked by A. movements of cattle within a 10-year timeframe, B. spatial proximity <2km and C. spatial proximity of <5km (all dark grey), and expected SNP differences from 10^4 simulations of the null hypothesis of no association between presence of a link and genetic similarity (light grey). Bars show the intervals containing 95% of the results from the null simulations

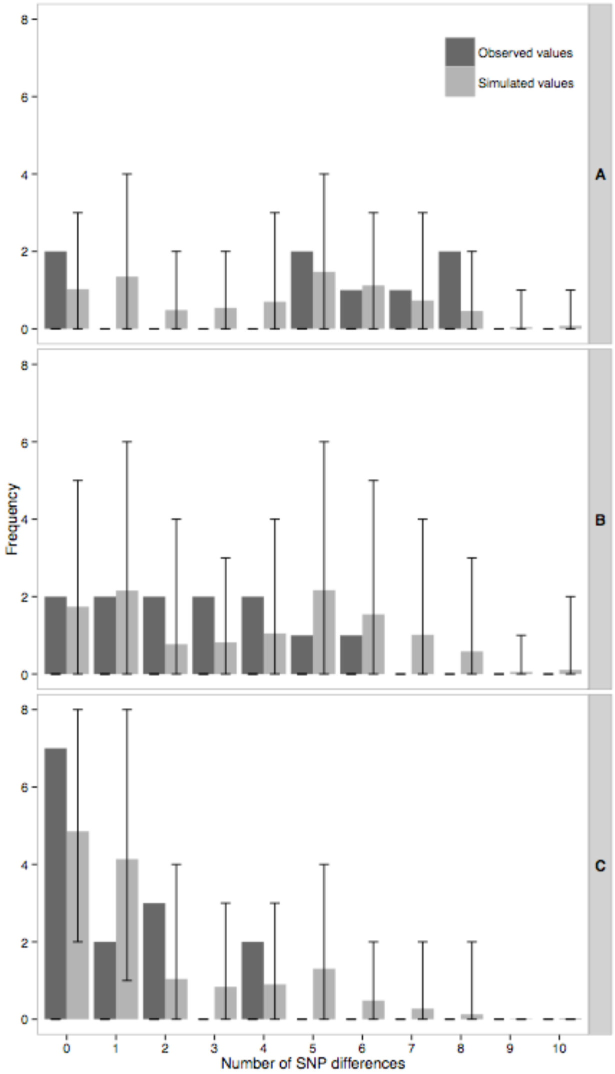

Figure S6: Map showing recorded cattle movement links (black arrows) and spatial relationships between cattle premises with VNTR-10 isolates sequenced in this study. Group 1 premises shown in red, Group 2 in orange

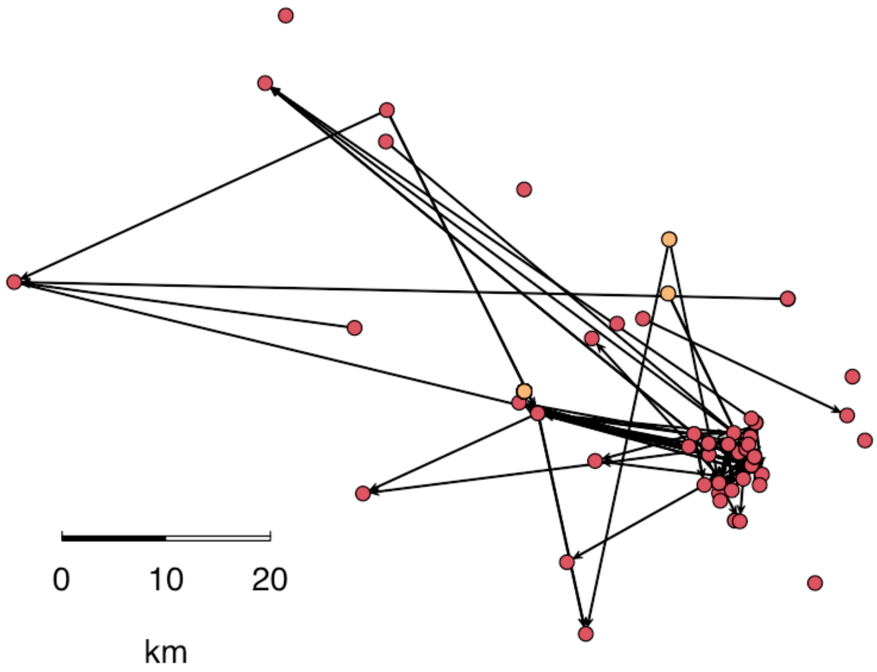

Figure S7: Histogram to show the distribution of branch-specific diffusion rates for terminal branches of the maximum clade credibility tree, estimated in BEAST under the Cauchy relaxed random walk model of continuous phylogeography. Superimposed line show a Cauchy distribution (scale=0.8, location=0)

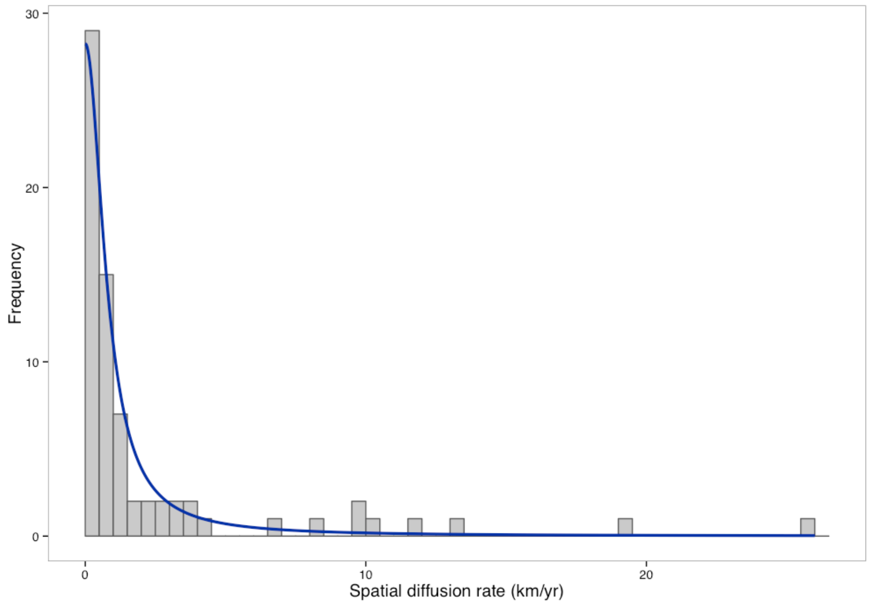

Figure S8: Comparison of branch time and distance travelled for terminal branches for the maximum clade credibility tree estimated under BEAST continuous phylogeography

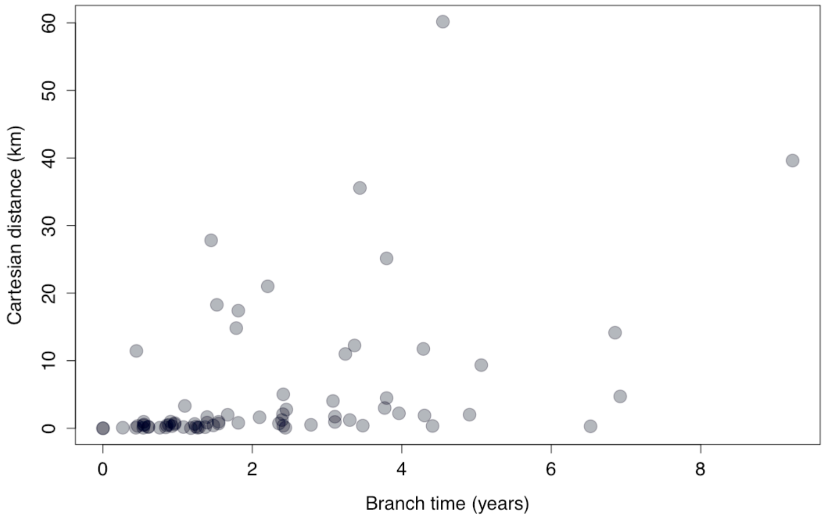

Figure S9: Violin plot of the posterior probabilities of diffusion rates recovered from BEAST phylogeographic analysis on data from 100 simulations of spatial diffusion along the time-stamped phylogeny generated from the Group 1 sequences. Red line indicates the diffusion rate that was simulated originally.

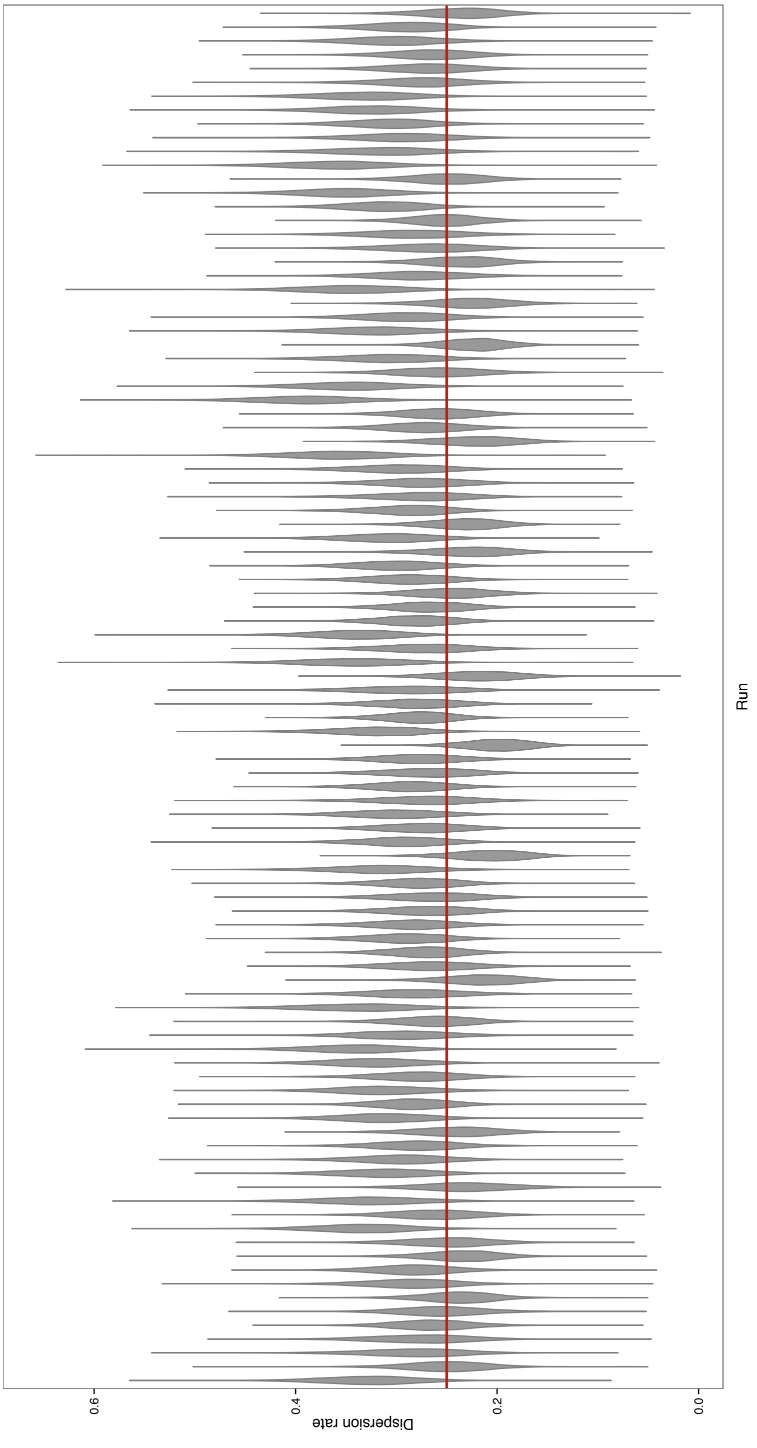

Table S1: Accession details and metadata for the raw reads sequences generated for this study.

Sample ID	Study accession no.	Sample accession no.	Label	VNTR type	Year	Host	Anonymised herd ID	Anonymised breakdown ID	Used in main analysis?
ID01	ERP001418	ERS137982		10	2004	Badger			Yes
ID02	ERP001418	ERS137977		10	2005	Badger			Yes
ID03	ERP001418	ERS137976		10	2005	Badger			Yes
ID04	ERP001418	ERS137974		10	2006	Badger			Yes
ID05	ERP001418	ERS137978		10	2007	Badger			Yes
ID06	ERP001418	ERS137975		10	1999	Cow	1	22960	Yes
ID07	ERP001418	ERS137973		10	1999	Cow	2	23171	Yes
ID08	ERP001418	ERS137981		10	1999	Cow	3	23483	Yes
ID10	ERP001418	ERS137979		10	2003	Cow	4	22243	Yes
ID11	ERP001418	ERS137980		10	2004	Cow	3	23481	Yes
ID12	ERP001418	ERS137972		10	2004	Cow	1	22957	Yes
ID13	ERP001418	ERS137992		10	2007	Cow	3	23480	
ID14	ERP001418	ERS137988		10	2007	Cow	3	23480	Yes
ID15	ERP001418	ERS137987		10	2007	Cow	3	23480	
ID16	ERP001418	ERS137985		10	2007	Cow	3	23480	
ID17	ERP001418	ERS137989		10	2008	Cow	5	2	
ID18	ERP001418	ERS137986		10	2008	Cow	5	2	
ID19	ERP001418	ERS137983		10	2008	Cow	5	2	
ID20	ERP001418	ERS137991		10	2008	Cow	5	2	Yes
ID21	ERP001418	ERS137990		10	2010	Cow	3	23479	Yes
ID22	ERP001418	ERS137984		10	2010	Cow	3	23479	
ID23	ERP001418	ERS138002		10	2010	Cow	3	23479	
ID24	ERP001418	ERS137999		10	2010	Cow	3	23479	
ID25	ERP001418	ERS137998		10	2010	Cow	3	23479	
ID26	ERP001418	ERS137996		10	2010	Cow	3	23479	
ID27	ERP001418	ERS138000		10	2010	Cow	5	1	
ID28	ERP001418	ERS137997		10	2010	Cow	5	1	Yes
ID29	ERP001418	ERS137994		10	2010	Cow	5	1	
ID30	ERP001418	ERS138001		10	2010	Cow	5	1	
ID31	ERP001418	ERS137993		10	2010	Cow	5	1	
ID32	ERP001418	ERS137995		10	2010	Cow	5	1	
ID33	PRJEB9025	ERS696617		10	2007	Badger			Yes
ID34	PRJEB9025	ERS696596		10	1998	Cow	39	22140	
ID35	PRJEB9025	ERS696614		10	1998	Cow	39	22140	
ID36	PRJEB9025	ERS696603		10	1999	Cow	43	21718	Yes
ID37	PRJEB9025	ERS696604		10	2000	Cow	37	22578	Yes
ID38	PRJEB9025	ERS696602		10	2000	Cow	16	19790	Yes
ID39	PRJEB9025	ERS696601		10	2000	Cow	12	24725	Yes
ID40	PRJEB9025	ERS696613		10	2003	Cow	34	24494	Yes
ID41	PRJEB9025	ERS696606		10	2003	Cow	45	476	
ID42	PRJEB9025	ERS696608		10	2003	Cow	45	476	Yes
ID43	PRJEB9025	ERS696609		10	2003	Cow	46	20725	Yes
ID44	PRJEB9025	ERS696605		10	2003	Cow	20	23730	Yes
ID45	PRJEB9025	ERS696611		10	2003	Cow	22	23638	Yes
ID46	PRJEB9025	ERS696600		10	2004	Cow	4	22243	
ID47	PRJEB9025	ERS696597		10	2004	Cow	48	20074	Yes
ID48	PRJEB9025	ERS696607		10	2004	Cow	38	22194	
ID49	PRJEB9025	ERS696599		10	2004	Cow	9	25002	Yes
ID50	PRJEB9025	ERS696616		10	2004	Cow	33	24501	Yes
ID51	PRJEB9025	ERS696595		10	2008	Cow	13	22461	Yes
ID52	PRJEB9025	ERS696598		10	2005	Cow	35	22742	Yes
ID53	PRJEB9025	ERS696618	2	10	2005	Cow	6	24858	Yes

ID54	PRJEB9025	ERS696610		10	2005	Cow	25	22485	
ID55	PRJEB9025	ERS696615		10	2005	Cow	25	22485	Yes
ID56	PRJEB9025	ERS696612		10	2005	Cow	39	22137	Yes
ID57	PRJEB9025	ERS696640		10	2006	Cow	36	22728	Yes
ID58	PRJEB9025	ERS696620		10	2006	Cow	25	22485	
ID59	PRJEB9025	ERS696637		10	2006	Cow	25	22485	
ID60	PRJEB9025	ERS696627		10	2006	Cow	29	40817	Yes
ID61	PRJEB9025	ERS696628		10	2006	Cow	10	24851	Yes
ID62	PRJEB9025	ERS696626		10	2006	Cow	10	24851	
ID63	PRJEB9025	ERS696625		10	2006	Cow	32	24630	Yes
ID64	PRJEB9025	ERS696636		10	2006	Cow	10	24851	
ID65	PRJEB9025	ERS696630		10	2006	Cow	10	24851	
ID66	PRJEB9025	ERS696632		10	2006	Cow	32	24630	
ID67	PRJEB9025	ERS696633		10	2007	Cow	23	22923	Yes
ID68	PRJEB9025	ERS696629		10	2007	Cow	25	22484	Yes
ID69	PRJEB9025	ERS696624		10	2007	Cow	17	40991	Yes
ID70	PRJEB9025	ERS696621		10	2007	Cow	13	22462	
ID71	PRJEB9025	ERS696631		10	2008	Cow	30	38551	Yes
ID72	PRJEB9025	ERS696623		10	2008	Cow	38	22193	Yes
ID73	PRJEB9025	ERS696639		10	2008	Cow	25	22483	Yes
ID74	PRJEB9025	ERS696619		10	2009	Cow	19	23968	Yes
ID75	PRJEB9025	ERS696622		10	2009	Cow	31	38045	Yes
ID76	PRJEB9025	ERS696641		10	2009	Cow	44	21508	Yes
ID77	PRJEB9025	ERS696634		10	2009	Cow	28	20439	Yes
ID78	PRJEB9025	ERS696638		10	2009	Cow	40	22039	Yes
ID79	PRJEB9025	ERS696635		10	2009	Cow	18	7802	Yes
ID80	PRJEB9025	ERS696663		10	2010	Cow	7	39898	Yes
ID81	PRJEB9025	ERS696643		10	2010	Cow	20	23726	Yes
ID82	PRJEB9025	ERS696660		10	2010	Cow	20	23726	
ID83	PRJEB9025	ERS696650		10	2010	Cow	20	23726	
ID84	PRJEB9025	ERS696651		10	2010	Cow	20	23726	
ID85	PRJEB9025	ERS696649		10	2010	Cow	20	23726	
ID86	PRJEB9025	ERS696648		10	2010	Cow	27	20514	
ID87	PRJEB9025	ERS696659		10	2010	Cow	27	20514	
ID88	PRJEB9025	ERS696653		10	2010	Cow	27	20514	
ID89	PRJEB9025	ERS696655	3	10	2010	Cow	27	20514	Yes
ID90	PRJEB9025	ERS696656		10	2010	Cow	27	20514	
ID91	PRJEB9025	ERS696652		10	2010	Cow	27	20514	
ID92	PRJEB9025	ERS696658		10	2010	Cow	27	20514	
ID93	PRJEB9025	ERS696647		10	2010	Cow	27	20514	
ID94	PRJEB9025	ERS696644		10	2010	Cow	27	20514	
ID95	PRJEB9025	ERS696654		10	2010	Cow	27	20514	
ID96	PRJEB9025	ERS696646		10	2010	Cow	27	20514	
ID97	PRJEB9025	ERS696662		10	2010	Cow	27	20514	
ID98	PRJEB9025	ERS696642		10	2010	Cow	27	20514	
ID99	PRJEB9025	ERS696645		10	2010	Cow	27	20514	
ID100	PRJEB9025	ERS696664		10	2010	Cow	21	23666	Yes
ID101	PRJEB9025	ERS696657		10	2010	Cow	20	23726	
ID102	PRJEB9025	ERS696661		10	2010	Cow	20	23726	
ID103	PRJEB9025	ERS696687		10	2010	Cow	27	20514	
ID104	PRJEB9025	ERS696666		10	2010	Cow	50	24037	Yes
ID105	PRJEB9025	ERS696684		10	2010	Cow	26	21133	Yes
ID106	PRJEB9025	ERS696673		10	2010	Cow	49	24077	Yes
ID107	PRJEB9025	ERS696674		10	2010	Cow	27	20513	
ID108	PRJEB9025	ERS696672		10	2011	Cow	51	23582	
ID109	PRJEB9025	ERS696671		10	2011	Cow	51	23582	
ID110	PRJEB9025	ERS696683		10	2011	Cow	51	23582	Yes
ID111	PRJEB9025	ERS696676		10	2011	Cow	51	23582	

ID112	PRJEB9025	ERS696678		10	2011	Cow	14	21118	Yes
ID113	PRJEB9025	ERS696679		10	2011	Cow	51	23582	
ID114	PRJEB9025	ERS696675		10	2011	Cow	51	23582	
ID115	PRJEB9025	ERS696681		10	2011	Cow	51	23582	
ID116	PRJEB9025	ERS696670		10	2011	Cow	51	23582	
ID117	PRJEB9025	ERS696667		10	2011	Cow	51	23582	
ID118	PRJEB9025	ERS696677		10	2011	Cow	51	23582	
ID119	PRJEB9025	ERS696669		10	2011	Cow	51	23582	
ID120	PRJEB9025	ERS696686		10	2011	Cow	51	23582	
ID121	PRJEB9025	ERS696665		10	2011	Cow	33	24499	Yes
ID122	PRJEB9025	ERS696668	1	10	2011	Cow	11	24827	Yes
ID123	PRJEB9025	ERS696680		10	2011	Cow	1	22955	Yes
ID124	PRJEB9025	ERS696685	A	1	2011	Cow	15	20860	Yes
ID125	PRJEB9025	ERS696682		4	2011	Cow	24	22731	Yes
ID126	PRJEB9025	ERS696710		10	2011	Cow	51	23582	
ID127	PRJEB9025	ERS696689		10	2011	Cow	51	23582	
ID128	PRJEB9025	ERS696707		10	1998	Cow	39	22140	Yes
ID130	PRJEB9025	ERS696696		10	2002	Cow	52	23267	Yes
ID131	PRJEB9025	ERS696697		10	2002	Cow	38	22194	Yes
ID132	PRJEB9025	ERS696695		10	2003	Cow	41	21975	Yes
ID133	PRJEB9025	ERS696694		10	2003	Cow	33	24503	Yes
ID134	PRJEB9025	ERS696706		10	2003	Cow	8	41335	Yes
ID135	PRJEB9025	ERS696699		10	2004	Cow	38	22194	
ID136	PRJEB9025	ERS696701		10	2004	Cow	46	20725	Yes
ID137	PRJEB9025	ERS696702		10	2004	Cow	16	19789	Yes
ID138	PRJEB9025	ERS696698		10	2005	Cow	42	21865	Yes
ID139	PRJEB9025	ERS696704		10	2005	Cow	36	22728	Yes
ID140	PRJEB9025	ERS696693		10	2007	Cow	13	22462	Yes
ID141	PRJEB9025	ERS696690		10	2010	Cow	49	24077	
ID142	PRJEB9025	ERS696700		10	2011	Cow	33	24499	
ID143	PRJEB9025	ERS696692		10	2011	Cow	33	24499	
ID144	PRJEB9025	ERS696709		10	2011	Cow	33	24499	
ID145	PRJEB9025	ERS696688		10	2011	Cow	33	24499	
ID146	PRJEB9025	ERS696691		10	2011	Cow	33	24499	
ID147	PRJEB9025	ERS696703		10	2011	Cow	33	24499	
ID148	PRJEB9025	ERS696708		10	2011	Cow	49	24076	Yes
ID149	PRJEB9025	ERS696705		10	2003	Cow	47	20552	Yes
ID150	PRJEB9025	ERS696711	B	1		Badger			Yes
ID151	PRJEB9025	ERS696712	C	1		Badger			Yes
ID152	PRJEB9025	ERS696713	D	1		Badger			Yes

Table S2: VNTR genotypes for VNTRs-1, 4 and 10 expressed as number of copies present at seven VNTR-loci

	VNTR locus						
	MV2163B QUB11B	MV4052 QUB26A	MV2461 ETR B	MV1895 QUB1895	MV2165 ETR A	MV2163 QUB11A	MV3232 QUB3232
VNTR-4	4	4	5	4	7	11	**7**
VNTR-1	**4**	4	5	4	7	11	**9**
VNTR-10	**3**	4	5	4	7	11	9

Table S3: Filter criteria used to identify high quality genetic variant sites in the bioinformatics workflow. "Total depth of coverage" refers to the minimum depth of coverage requirement, while "Depth of coverage on each strand" refers to the minimum depth of coverage required on each strand (filter aimed at reducing strand bias).

	Batch 1		Batch 2		Batch 3	
	Strict	Relaxed	Strict	Relaxed	Strict	Relaxed
Total depth of coverage	50	14	25	7	25	5
Depth of coverage on each strand	6	2	2	1	2	1
Mapping quality	35	20	35	20	40	35
Homozygosity	95%	95%	95%	95%	95%	95%

Table S4: Statistical comparison of VNTR-10 herd breakdowns to: A. breakdowns attributable to other strains in the local area; and B. bTB breakdowns attributable to other strains across the whole of NI.

**Variable**	**Description**	**A.** Local herds			**B.** All herds		
		t	***df***	***P***	***t***	***df***	***P***
**Herd size**	Number of animals in the herd at the beginning of the breakdown year	-1.83	99.63	0.07	-2.53	93.85	0.013
**Neighbours**	Number of active herds within 1km radius of the herd during the previous year	2.96	102.09	0.004	9.36	95.55	<0.001
**Neighbours with bTB**	Number of herds within a 1km radius with bTB reactors during the previous year	0.97	103.99	0.332	0.1	94.51	0.923
**Sett density**	Mean density of active main badger setts per km^2	1.56	100.99	0.122	-11.06	94.28	<0.001
**Badger density**	Mean density of badgers per km^2	1.82	102.4	0.072	-8.8	94.76	<0.001
		Chi.sq	***df***	***P***	***Chi.sq***	***df***	***P***
**Herd type**	Classified as beef if ≥ 50% animals are from beef breeds	1.15	1	0.284	0.87	1	0.35
**bTB history**	bTB in the herd within the 24 months prior to the breakdown	0.76	1	0.383	2.59	1	0.10 7
**Imports from RoI**	Animals imported into the herd from the Republic of Ireland during the previous year	1.91	1	0.167	0	1	1
